# Independent and interactive effects of eye rubbing and atopy on keratoconus

**DOI:** 10.3389/fimmu.2022.999435

**Published:** 2022-09-29

**Authors:** Kaili Yang, Dan Li, Liyan Xu, Chenjiu Pang, Dongqing Zhao, Shengwei Ren

**Affiliations:** Henan Provincial People’s Hospital, Henan Eye Hospital, Henan Eye Institute, People’s Hospital of Zhengzhou University, Henan University People’s Hospital, Zhengzhou, China

**Keywords:** eye rubbing, atopy, keratoconus, multiplicative interaction, additive interaction

## Abstract

**Purpose:**

To evaluate the independent and interactive effects of eye rubbing and atopy on keratoconus (KC) in central China.

**Methods:**

A total of 330 KC patients and 330 controls were recruited in the case-control study. Eye rubbing and history of atopy were recorded through face-to-face interviews. The association between KC and eye rubbing, atopy, interactive effects of eye rubbing and atopy were analyzed by logistic regression, and the odds ratios (*OR*), relative excess risk due to interaction (RERI), attributable proportion (AP), synergy (S) index, and 95% confidence intervals (95% *CI*) were calculated.

**Results:**

A total of 228 patients (69.09%) had an eye rubbing history, and 53 (16.06%) had an atopy history in the KC group, which were both higher than that in the control group (p<0.001). Eye rubbing and atopy were positively associated with KC in multivariate analysis, with ORs (95% CIs) of 15.11 (10.02, 22.80) and 5.30 (2.59, 10.84), respectively. Compared to non-eye rubbing and non-atopy eyes, the risk for eye rubbing coexisted with atopy was 52.31 (12.25, 223.35). No significant associations were found between KC and multiplicative interaction (p=0.608). The RERI, AP, and S values were 32.89 (-43.35, 109.14), 0.63 (0.05, 1.21), and 2.79 (0.56, 13.96), respectively, with no significant association between additive interaction and KC. No significant associations were found between eye rubbing, atopy and the severity of KC (p>0.05).

**Conclusion:**

Eye rubbing and atopy were separately positively associated with KC, and there was a strong impact of coexistent eye rubbing and atopy on KC in China. Further multi-center and cohort study need to be conducted to explore the role of eye rubbing and atopy in the occurrence and development of KC.

## Introduction

Keratoconus (KC) is a complex corneal disorder characterized by progressive thinning and cone-shaped protrusion of the cornea (1, 2). A recent review reported that the prevalence of KC ranged from 0.2 to 4 790 per 100,000 worldwide (3). KC is usually accompanied by varying degrees of visual impairment, leading to blindness in severe cases (4–6). The exact etiology of KC is still unclear. Multiple genetic studies have indicated that genetic components play important roles in KC (3, 7, 8). In addition, environmental factors, which include age, gender, ethnicity, ultraviolet exposure, atopy, and eye rubbing, have been recognized to be associated with KC (3, 7, 9, 10). Identifying and controlling the modifiable risk factors of KC would help reduce the incidence of KC, and further decrease its economic burden for the society (9, 11).

Advising KC patients to stop eye rubbing has been a common recommendation, and the association between eye rubbing and KC has been widely studied in the past (12–15). Some cases of unilateral KC have been reported to have developed in response to a unilateral habit of eye rubbing, which indicated that there might exist a strong association between eye rubbing and KC (16–20). In addition, Sahebjadah et al. (13) conducted a meta-analysis including six case-control studies and found that eye rubbing was positively associated with KC. However, a recent meta-analysis reported no significant association between eye rubbing and KC, with one prospective cohort study, three cross-sectional studies, and seven case-control studies (14). The heterogeneity between different studies prompted researchers to further explore the association between eye rubbing and KC.

Allergic conjunctivitis, which result in a chronic inflammation of the ocular surface, was found to be associated with KC (21). In addition, atopy is an important condition for KC, and the association between atopy and KC has been evaluated for many years (9, 22). Although atopy has been suggested as a risk factor for KC (23), several recent studies failed to demonstrate the association (4, 14). Multifactor analysis, including eye rubbing and atopy, showed that eye rubbing was still a risk factor for KC (14, 24, 25). In contrast, the association between atopy and KC was inconsistent (14, 24, 25). In addition, the report on the joint effects of eye rubbing and atopy on the development of KC was limited. Therefore, the present study aimed to evaluate the independent effect of eye rubbing and atopy on the development and severity of KC in central China and further explore the association between KC and interactive effect of eye rubbing and atopy, which would provide references for the clinical management of KC.

## Methods

### Study subjects

This case-control study included subjects visiting Henan Eye Hospital from January 2019 to January 2022. KC was diagnosed based on the following criteria (1, 2, 26): at least one positive sign on slit-lamp examination (Fleischer’s ring, Vogt’s striae, Munson’s sign, or corneal scar), an asymmetric bowtie pattern with or without skewed axes and Belin Ambrosio enhanced ectasia total deviation index (BAD) value > 2.6 revealed by a corneal topography map. Participants scheduled for refractive surgery with spherical equivalent (SPH)<8.00 diopters (D), corneal astigmatism (CYL)<1.50 D, and corrected distance visual acuity (CDVA) in LogMAR ≤ 0.1 were included in the control group. The exclusion criteria for both groups were patients with diabetes and a positive family KC history, eye with a rigid contact lens used in the last four weeks or a soft contact lens used in the last two weeks, eyes with other ocular diseases (cataract, glaucoma, and fundi conditions) or any ocular surgery history, and eye with an anterior stromal scar. Finally, 330 KC eyes (330 patients) and 330 control eyes (330 subjects), matched with the principles of age (3 years) and sex, were recruited in the present study. The study protocol was approved by the institutional review board of our institute (HNEECKY-2019 (5)), and all the procedures followed the guidelines of the Declaration of Helsinki.

### Clinical examination data

Demographic characteristics, eye rubbing, and atopy history were recorded through a face-to-face interview (27). Eye rubbing is a common activity occurring at different times of the day: upon waking, before sleep, during extended computer work, and throughout the day in response to ocular itching and irritation (28, 29). The eye rubbing habit is usually benign, but when it is performed too vigorously or too frequently, it becomes pathological, damaging the cornea (20). The frequency of eye rubbing was obtained by asking subjects how often they rubbed their eyes, and eye rubbing in the current analysis was defined as a frequency greater than or equal to once daily (30). In addition, the atopy was defined as a history of clinically relevant sensitization to pollen, house dust mite, or animal hair related to allergic rhinoconjunctivitis, or the presence of allergic asthma or atopic dermatitis (31).

Experienced operators performed the slit-lamp and ophthalmoscope examinations. The SPH, CYL, and CDVA were obtained through objective refraction (Topcon KR-800) and subjective refraction (Topcon CV-5000), and the intraocular pressure (IOP) was measured by a non-contact tonometer (Topcon, Japan). Corneal tomographic parameters were performed by Pentacam HR software, and steep keratometry (Ks), flat keratometry (Kf), the max keratometry (Kmax), and thinnest corneal thickness (TCT) values were recorded. The severity of KC was classified as early KC (TKC<2), moderate KC (2≤TKC<3), and advanced KC (TKC≥3) (32).

### Statistical analysis

Quantitative data were shown in means and standard deviations (SD), and qualitative data were presented as frequencies with percentages. Evaluation of significant differences between KC and control, eye rubbing and non-eye rubbing, atopy, and non-atopy groups was performed with a two-sample t-test and a chi-squared test. The effects of eye rubbing, atopy, and eye rubbing coexisted atopy were analyzed using logistic regression. The multiplicative interaction of eye rubbing and atopy was investigated by adding an “eye rubbing _*_ atopy” term into the logistic regression model, and the odds ratios (*OR*) and 95% confidence intervals (95% *CI*) of the term was recoded. The additive interaction was investigated by calculating the relative excess risk due to interaction (RERI), the attributable proportion due to interaction (AP) and synergy (S) index according to the algorism released by Andersson et al. (33) For RERI and AP, a 95% *CI* not including 0 indicated significance; and for S, a 95% *CI* not including 1 represented significance. All the statistical analyses were performed by SPSS 23.0 (IBM, USA), and p<0.05 (two-tailed test) was considered a significant difference.

## Results

### Clinical data of KC and control groups

The KC group included 238 males and 92 females, and the control group included 223 males and 107 females (p*=*0.203, **Table 1**). The mean age of the KC group was 21.12 ± 5.22 years, with 20.89 ± 4.72 years in the control group (p*=*0.553). KC eyes had higher values of SPH, CYL, CDVA (logMAR), Kf, Ks, Kmax, and lower values of IOP, TCT than control eyes (p<0.001). There were 228 eyes (69.09%) with a history of eye rubbing in the KC group, higher than that in the control group (13.93%, p<0.001). There were 53 eyes (16.06%) with a history of atopy in the KC group, higher than that in the control group (4.85%, p<0.001).

**Table 1:** Demographic data in control and KC eyes.

Parameters	Control (N=330)	KC (N=330)	χ^2^/t	*P*
Age(year)	20.89 ± 4.72	21.12 ± 5.22	-0.594	0.553
Gender, N(%)			1.619	0.203
Male	223 (67.58)	238 (72.12)		
Female	107 (32.42)	92 (27.88)		
SPH(D)	-4.48 ± 1.75	-5.12 ± 3.96	2.595	0.010
CYL(D)	-0.70 ± 0.65	-4.03 ± 2.48	22.493	<0.001
CDVA (logMAR)	0.01 ± 0.02	0.36 ± 0.29	-21.286	<0.001
IOP (mmHg)	15.51 ± 2.53	13.89 ± 4.68	5.461	<0.001
Kf (D)	42.26 ± 1.42	47.34 ± 4.55	-19.299	<0.001
Ks (D)	43.52 ± 1.60	51.18 ± 5.32	-24.993	<0.001
Kmax (D)	44.16 ± 1.66	58.10 ± 8.41	-28.849	<0.001
TCT (um)	543.21 ± 30.64	458.93 ± 40.85	29.954	<0.001
Eye rubbing, N (%)			206.704	<0.001
No	284 (86.06)	102 (30.91)		
Yes	46 (13.93)	228 (69.09)		
Atopy, N (%)			22.157	<0.001
No	314 (95.15)	277 (83.94)		
Yes	16 (4.85)	53 (16.06)		

KC, keratoconus; SPH, spherical equivalent; CYL, corneal astigmatism; CDVA, corrected distance visual acuity; IOP, intraocular pressure; Kf, flat keratometry; Ks, steep keratometry; Kmax, max keratometry; TCT, thinnest corneal thickness.

### Logistic regression analysis

Univariate logistic regression indicated that eye rubbing (model 1) and atopy (model 2) were positively associated with KC, and the *ORs* (95% *CIs*) were 13.80 (9.35, 20.37) and 3.76 (2.10, 6.72), respectively. Multivariate analysis (model 3) showed that positive associations still existed with *OR* (95% *CI*) values of 15.11 (10.02, 22.80) for eye rubbing and 5.30 (2.59, 10.84) for atopy, respectively (**Table 2**).

**Table 2:** Logistic regression results of eye rubbing and atopy on KC.

Parameters	b	Stand error	χ^2^	*P*	*OR* (95%*CI*)
**Model 1**					
Eye rubbing	2.625	0.199	174.627	<0.001	13.8 (9.35,20.37)
**Model 2**					
Atopy	1.323	0.297	19.856	<0.001	3.76 (2.10,6.72)
**Model 3**					
Eye rubbing	2.715	0.210	167.551	<0.001	15.11 (10.02,22.8)
Atopy	1.668	0.365	20.915	<0.001	5.30 (2.59,10.84)
Eye rubbing_*_Atopy	-0.427	0.833	0.262	0.608	0.65 (0.13,3.34)

Model 1, univariate regression involving eye rubbing.

Model 2, univariate regression involving atopy.

Model 3, multivariate regression involving eye rubbing, atopy and eye rubbing_*_atopy.

### Interactive effects of eye rubbing and atopy

The multiplicative effect of eye rubbing and atopy was found not to be associated with KC (*OR*:0.65, 95% *CI*: 0.13 to 3.34, p=0.608). **Table 3** shows the association between the joint effect of eye rubbing and atopy and KC. Compared to non-eye rubbing and non-atopy eyes, the *OR* (95% *CI*) for eye rubbing coexisted with atopy was 52.31 (12.25, 223.35). The RERI, AP and S were 32.89 (95% *CI*: -43.35 to 109.14), 0.63 (95% *CI*: 0.05 to 1.21), and 2.79 (95% *CI*: 0.56 to 13.96), respectively.

**Table 3:** Joint effect of eye rubbing and atopy on KC.

Category	Eye rubbing	Atopy	KC	control	b	*OR* (95%*CI*)	*P*
1	–	–	80	270	–	1.00	
2	–	+	22	14	1.668	5.30 (2.59,10.84)	<0.001
3	+	–	197	44	2.715	15.11 (10.02,22.80)	<0.001
4	+	+	31	2	3.957	52.31 (12.25,223.35)	<0.001

### Eye rubbing, atopy, and KC severity

**Figure 1:**
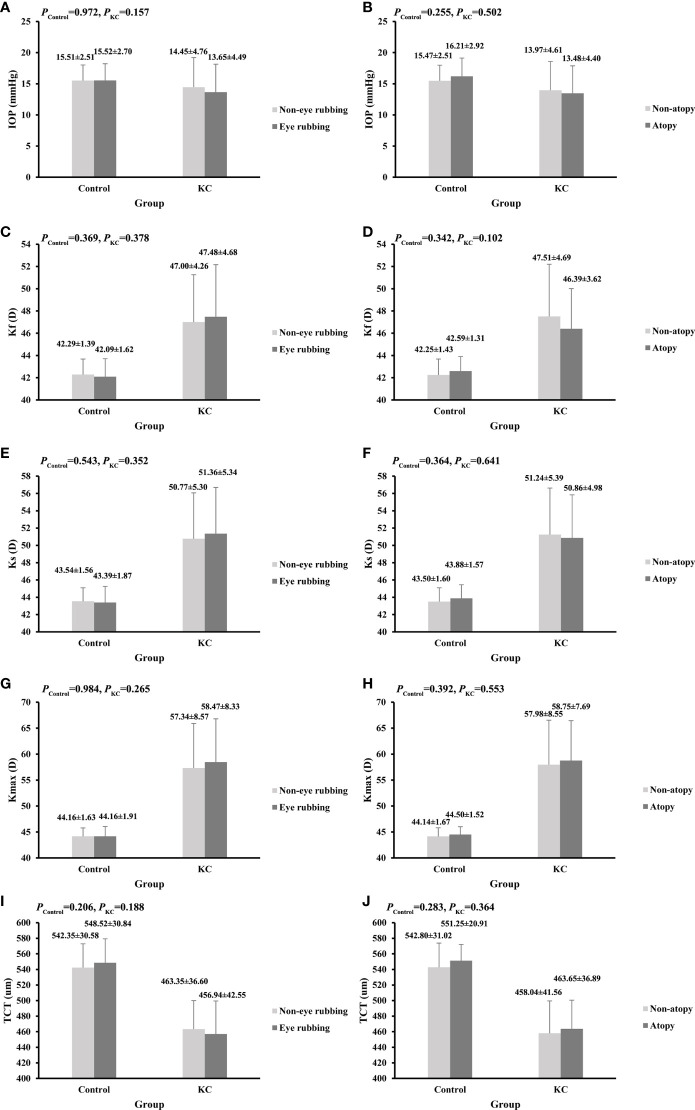
Comparisons of corneal parameters in KC and control groups. **(A)** IOP between eye rubbing and non-eye rubbing; **(B)** IOP between atopy and non-atopy; **(C)** Kf between eye rubbing and non-eye rubbing; **(D)** Kf between atopy and non-atopy; **(E)** Ks between eye rubbing and non-eye rubbing; **(F)** Ks between atopy and non-atopy; **(G)** Kmax between eye rubbing and non-eye rubbing;**(H)** Kmax between atopy and non-atopy; **(I)** TCT between eye rubbing and non-eye rubbing; **(J)** TCT between atopy and non-atopy.

**Figure 1** shows the comparisons of corneal parameters in eye rubbing vs. non-eye rubbing and atopy vs. non-atopy groups. Although no significant difference was found in the current analysis, KC eyes with a history of rubbing tend to have higher values of Ks, Kf, Kmax, and a lower TCT value than those without eye rubbing (p>0.05). In addition, no significant differences in KC severity were found in eye rubbing vs. non-eye rubbing, atopy vs. non-atopy, and different joint effects (p>0.05, **Table 4**).

**Table 4:** Association between eye rubbing, atopy and KC severity.

Category, N(%)	TKC <2	2 ≤TKC <3	TKC ≥3	χ^2^	*P*
**Eye rubbing**				2.866	0.239
No	28 (38.89)	38 (29.69)	36 (27.69)		
Yes	44 (61.11)	90 (70.31)	94 (72.31)		
**Atopy**				2.295	0.317
No	64 (88.90)	108 (84.40)	105 (80.80)		
Yes	8 (11.10)	20 (15.60)	25 (19.20)		
**Eye rubbing and atopy**				10.982	0.089
Category 1	23( 31.94)	27 (20.09)	30 (23.08)		
Category 2	5 (6.94)	11 (8.59)	6 (4.62)		
Category 3	41 (56.94)	81 (63.28)	75 (57.69)		
Category 4	3 (4.17)	9 (7.03)	19 (14.62)		

Category 1: eye rubbing (-) and atopy (-); Category 2: eye rubbing (-) and atopy (+); Category 3: eye rubbing (+) and atopy (-); Category 4: eye rubbing (+) and atopy (+).

## Discussion

A greater understanding of the risk factors for KC may allow for earlier diagnosis and, therefore, lower the incidence of KC (3, 7). The case-control study showed that eye rubbing and atopy were positive associated with KC, with no significant association with KC severity. In addition, eye rubbing coexisted with atopy was strong associated with KC, although no interactive effect of eye rubbing and atopy was found on KC.

KC is typically considered to be bilateral; however, some unilateral cases have been reported, which have been found to develop only in the eye subjected to frequent and abnormal episodes of rubbing trauma (18, 19). In addition, a series of case-control studies demonstrated that KC patients were more likely to rub their eyes (13). The pooled *OR* (95% *CI*) of eye rubbing with six case-control studies in a meta-analysis was 6.46 (4.12, 10.1) (13), with a mean range of 3.35 (2.35, 4.77) (27) to 10.15 (4.37, 23.54) (25). Similar positive associations have also been found in other studies, which was consistent to the current results (24, 34–36). Despite these claims, a recent meta-analysis by Seth et al. revealed no significant association between eye rubbing and KC with an *OR* (95% *CI*) of 1.59 (0.70, 3.63) (14). Furthermore, Owens et al. (37), Millodot et al. (23), and Moleiro AF et al. (22) reported that eye rubbing was not associated with KC. The study sample, ethnicity, populations heterogeneity, and definition of eye rubbing may explain the differences between studies, necessitating multicenter large-sample studies to verify the association in the future.

It was reported that the persistent corneal trauma caused by external forces such as eye rubbing may be an essential condition for the beginning and progression of KC (7). The role of eye rubbing has been explored for many years, and several mechanisms have been proposed to explain the association between eye rubbing and KC (13, 20, 38). Eye rubbing traumatizes the keratocytes, and subsequent fluctuations in IOP may cause local inflammation, leading to atrophy and KC pathogenesis (39, 40). McMonnies reported that eye rubbing could raise the corneal temperature, and the rubbing-related buckling and flexure of collagen fibrils may facilitate cone formation, changing the corneal biomechanical stability (38, 41). Furthermore, Gritz et al. (42) reported that eye rubbing might damage the epithelium, leading to cytokine release, myofibroblast differentiation, changes in the corneal shape, corneal biomechanical forces, and the thinning of the corneal tissue. Although eye rubbing has been widely explored in KC patients, the exact mechanisms of how eye rubbing is associated with KC are still unclear and should be further explored (13).

Atopy is defined as a combination of many conditions, such as allergy, asthma, atopic dermatitis, etc. (22, 24) Previous study reported that allergic conjunctivitis would result in a chronic inflammation of the ocular surface, and it has long been associated with KC (21). The association between atopy and KC has been reported since the beginning of the 20th century, and many conflicting reports have been published (23, 24). The present study showed that atopy was a risk factor for KC, consistent with a study by Millodot et al. (23) In contrast, no significant association has been demonstrated between atopy and KC in other studies (9, 14, 24). The variations in definitions, populations heterogeneity, and duration of atopy may arise from the conflicting results of different studies (25). Although not truly understood, inflammation and eye rubbing habits were the common explanations for the relationship between atopy and KC (4, 9). On one hand, atopy is a factor leading to the irritation of the eye, which could initiate eye rubbing behaviors and KC (9, 22). There was a common denominator between KC and atopic disease patients that many of them reported the habit of eye rubbing due to the pruritus around the eyelids as a symptom of their allergic disease (7). On the other hand, some believe that atopy in isolation plays the initiating role, and the corneal microenvironment in KC may be affected by the systemic inflammatory changes and oxidative stresses (14, 24). There are increasing evidences for the activity of the immune system in the pathogenesis of KC (43, 44). A recent population-based study found KC was positively associated with multiple immune-mediated diseases, which provided argument that systemic inflammatory responses may influence its onset (43). The relationship between atopy and KC is inconclusive and more extensive investigations are necessary in the future.

Previous studies have explored the association between eye rubbing, atopy, and KC (22, 45). As a common provocative factor for eye rubbing, atopy was found to be positively associated with KC (25). Increased inflammatory molecules and proteases and itching-related eye rubbing were thought to contribute to the development and progression of KC in atopic patients (46). The multifactor analysis in the present study showed that atopy and eye rubbing were separately associated with KC, consistent with a study by Gordon-Shaag et al. (25) However, several multifactor analyses indicated that eye rubbing was a risk factor for KC, while atopy was not (9, 14, 24). The variations in disease severity, the definition of atopy and rubbing, differences in the duration of atopy, and populations heterogeneity may explain the discrepancies in the results of different studies (9). In addition, the present study showed that eye rubbing coexisted with atopy had a much higher risk of KC than those with only one factor. However, the interactive effects of eye rubbing and atopy, including multiplicative and additive effects, were not associated with KC, necessitating more extensive investigations in the future. In addition, the etiology of KC is complex, the genetic factors play important roles besides environmental factors in the occurrence and progression of KC (3, 7). The levels of proteases and inflammatory mediators, which increased after eye rubbing and atopy, are regulated by the genetic polymorphisms (44). Thus, the comprehensive effect of eye rubbing, atopy, and genetic factors should be further explored in later.

Some previous studies have reported loose associations between eye rubbing, atopy, and KC severity (27, 47). The present study revealed that KC patients with eye rubbing had higher values of Ks, Kf, and Kmax, and a lower value of TCT than that of patients without eye rubbing. However, the differences were not significant, consistent with a study by Naderan et al., suggesting that eye rubbing might be associated with the progression of KC (27). In addition, Naderan et al. reported that KC patients with a higher frequency of eye rubbing had more severe KC (27). However, there was no significant association between eye rubbing and KC severity in the current study. A prospective cohort study showed that eye rubbing increased the irregularity index of the corneal surface, and 0.5 D of astigmatism was found to be induced after 60 s of eye rubbing (47). In addition, no significant difference in clinical parameters was found between atopy and non-atopy KC eyes, which was inconsistent with a previous study (48). Kaya et al. (48) reported that the TCT in KC eyes with atopy was lower than in KC eyes without atopy, with no significant difference in K reading. The study design and the heterogeneity of populations lead to the result inconsistent. Furthermore, no significant difference in KC severity between atopy and non-atopy KC eyes was found, which need further study to explore the results in later.

This case-control study demonstrated that eye rubbing and atopy were separately associated with KC, and eye rubbing coexisted with atopy was strong positively associated with KC. However, some limitations should be noticed. Firstly, the information on eye rubbing and atopy was obtained by questionnaire, which might be associated with recall bias. Therefore, the frequency of eye rubbing might be underestimated, despite carrying out all the steps through standardized procedures. Secondly, the subjects were from a tertiary center, in which most patients in central China choose to seek help. Although patients and controls were matched for age, sex, and location to give an unbiased estimate of population exposure, the extrapolation of the findings is limited and multicenter studies are needed for validation. Thirdly, the habit of eye rubbing has many aspects (49). The present study evaluated the association between the eye rubbing frequency and KC, while the force, duration, and methods (fingernail, the fingertip, and the knuckle) of eye rubbing that were regarded as important parameters, were not evaluated. Further research with higher quality methodology is necessary to elucidate the role of eye rubbing and atopy in the development and severity of KC.

## Conclusion

In conclusion, eye rubbing and atopy were positively associated with KC and not related to the KC severity in central China. Furthermore, the coexistence of eye rubbing and atopy was strong positively associated with KC. This study provides guidance for the clinical diagnosis and management of KC and is the basis for further research to deeply explore the role of eye rubbing and atopy in the incidence and development of KC.

## Data availability statement

The original contributions presented in the study are included in the article/supplementary material. Further inquiries can be directed to the corresponding author.

## Ethics statement

The studies involving human participants were reviewed and approved by Institutional Review Board of Henan Eye Hospital [ethical approval number: HNEECKY-2019 (5)]. Written informed consent to participate in this study was provided by the participants’ legal guardian/next of kin.

## Author contributions

KY: Writing- Original draft preparation, Methodology, Software. DL: Methodology, Data curation. LX: Methodology, Data curation, Investigation. CP: Editing and Paper revision. DZ: Visualization, Investigation. SR: Conceptualization, Writing-Reviewing and Editing.

## Funding

This research was supported by the National Natural Science Foundation of China (No. 81200664), Henan Provincial Medical Science Building Key Program (No. SBGJ202002028, SBGJ202102051), Henan Provincial Medical Science and Technology Joint Program (No. LHGJ20200066, LHGJ20210080), Open Program of Shandong Provincial Key Laboratory of Ophthalmology (No. 2018-04), Henan Young Health Science and Technology Innovation Outstanding Program (No. YXKC2020023), Henan Provincial Science and Technology Research Project (No. 222102310599, 222102310307), Special Program for Basic Research of Henan Eye Hospital (No. 20JCZD003), Youth Special Program for Basic Research of Henan Eye Hospital (No. 21JCQN006, 21JCQN008), Basic Research and Cultivation Foundation for Young Teachers of Zhengzhou University (No. JC202051049).

## Conflict of interest

The authors declare that the research was conducted in the absence of any commercial or financial relationships that could be construed as a potential conflict of interest.

## Publisher’s note

All claims expressed in this article are solely those of the authors and do not necessarily represent those of their affiliated organizations, or those of the publisher, the editors and the reviewers. Any product that may be evaluated in this article, or claim that may be made by its manufacturer, is not guaranteed or endorsed by the publisher.
